# Defining the heterogeneity of unbalanced structural variation underlying breast cancer susceptibility by nanopore genome sequencing

**DOI:** 10.1038/s41431-023-01284-1

**Published:** 2023-02-16

**Authors:** Katherine Dixon, Yaoqing Shen, Kieran O’Neill, Karen L. Mungall, Simon Chan, Steve Bilobram, Wei Zhang, Marjorie Bezeau, Alshanee Sharma, Alexandra Fok, Andrew J. Mungall, Richard Moore, Ian Bosdet, My Linh Thibodeau, Sophie Sun, Stephen Yip, Kasmintan A. Schrader, Steven J. M. Jones

**Affiliations:** 1 Department of Medical Genetics, University of British Columbia, Vancouver, BC Canada; 2 Canada’s Michael Smith Genome Sciences Centre, BC Cancer, Vancouver, BC Canada; 3 Hereditary Cancer Program, BC Cancer, Vancouver, BC Canada; 4 Department of Pathology and Laboratory Medicine, University of British Columbia, Vancouver, BC Canada; 5 Department of Medical Oncology, BC Cancer, Vancouver, BC Canada

**Keywords:** Sequencing, Genetic testing

## Abstract

Germline structural variants (SVs) are challenging to resolve by conventional genetic testing assays. Long-read sequencing has improved the global characterization of SVs, but its sensitivity at cancer susceptibility loci has not been reported. Nanopore long-read genome sequencing was performed for nineteen individuals with pathogenic copy number alterations in *BRCA1*, *BRCA2*, *CHEK2* and *PALB2* identified by prior clinical testing. Fourteen variants, which spanned single exons to whole genes and included a tandem duplication, were accurately represented. Defining the precise breakpoints of SVs in *BRCA1* and *CHEK2* revealed unforeseen allelic heterogeneity and informed the mechanisms underlying the formation of recurrent deletions. Integrating read-based and statistical phasing further helped define extended haplotypes associated with founder alleles. Long-read sequencing is a sensitive method for characterizing private, recurrent and founder SVs underlying breast cancer susceptibility. Our findings demonstrate the potential for nanopore sequencing as a powerful genetic testing assay in the hereditary cancer setting.

## Introduction

Breast cancer is the most common cancer in females with an estimated 2.3 million new diagnoses worldwide in 2020 [1]. Rare variants in genes associated with high-penetrance cancer predisposition syndromes confer a strong genetic susceptibility in around 5–10% of cases depending on ascertainment criteria. *BRCA1*, *BRCA2*, and the moderate-penetrance genes *CHEK2* and *PALB2* have also been associated with increased risks for male breast cancer, a rare disease in which 18% of cases may be related to clinically actionable germline variants [2, 3]. Identifying carriers for moderate- to high-penetrance variants can improve clinical outcomes by informing disease risk and prognosis, and guide recommendations for prophylactic intervention, cancer screening and therapy. However, many individuals who undergo genetic testing based on a strong personal or family history of breast and other syndrome-related cancers receive uninformative results [4].

Short-read sequencing (SRS) is the most common technology used in clinical laboratories for genetic testing due to its high throughput, high analytic validity, and low relative cost. Despite these advantages, one in seven pathogenic germline variants are challenging to detect using SRS [5]. Long-read sequencing (LRS) has shown potential for improving rates of molecular diagnosis by more accurately identifying structural variants (SVs) and repeat expansions, resolving complex rearrangements, and informing phase of candidate variants [6]. Although the clinical utility of LRS has been described in the diagnosis of various genetic syndromes, the sensitivity of long-read technologies for characterizing structural variation at loci commonly tested in the clinical setting is not well-established.

Here, we assessed the accuracy of nanopore long-read genome sequencing (GS) for characterizing pathogenic germline SVs in four breast cancer susceptibility genes. Expanding upon results from clinical testing, precise breakpoints could be defined at nucleotide resolution, revealing uncharacterized allelic heterogeneity at the loci of recurrent and founder variants. Our findings may inform the future implementation of LRS as an alternative to standard clinical assays.

## Materials and methods

This study was approved by the University of British Columbia Clinical Research Ethics Board (H19-01594). All participants provided written informed consent. PCR-free genome libraries were prepared from DNA isolated from peripheral blood lymphocytes and sequenced on the Oxford Nanopore Technologies PromethION. Single nucleotide variant (SNV) and small insertion and deletion (indel) calling and phasing were performed using an established pipeline [7]. SVs were manually reviewed in IGV. SV breakpoints were defined by local assembly-derived contigs where possible, and reported according to HGVS sequence variant nomenclature. Haplotype inference was performed using integrated read- and population-based phasing [8]. Please refer to the Supplementary Materials for detailed methods.

## Results

GS was performed for 19 individuals from 18 families with pathogenic deletions and duplications in *BRCA1*, *BRCA2*, *CHEK2* or *PALB2* identified by prior clinical testing (Supplementary Table S1). Individuals were referred for index or carrier testing on the basis of a suspected inherited predisposition to breast cancer or known familial variant, respectively. Sequencing was performed to a median coverage of 21.6X (13.1–36.5X), achieving a median read N50 of 14.5 kb (6.57–23.5 kb) (Supplementary Table S2).

**Fig. 1:**
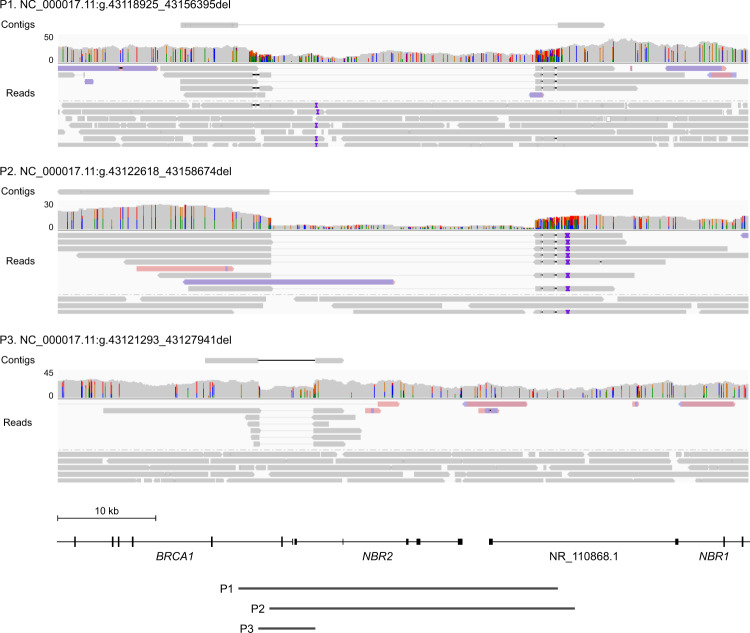
Recurrent deletions of *BRCA1* exons 1–2 are mediated by sequence homology between *BRCA1* intron 2 and adjacent loci. Read alignments and contigs derived by local assembly of variant supporting reads at the *BRCA1* locus. Telomeric breakpoints within intron 1 of the *BRCA1* pseudogene were associated with deletions of 37,470 bp (P1) and 36,056 bp (P2), and breakpoints within *NBR2* intron 1 were associated with a deletion of 6649 bp (P3). For clarity, mismatch bases and indels <50 bp are not shown.

Variants in all 19 carriers, representing 14 distinct SVs ranging in size from 510 bp to 108 kb, were detected by nanopore sequencing (Table 1). While some variants were not identified by agnostic SV calling, all known SVs were supported by at least three reads (Supplementary Table S2). The precise breakpoints were refined in all but one case with low coverage and low complexity sequence at one breakpoint. Previously unknown allelic heterogeneity was revealed at the locus of *BRCA1*: three deletions spanning *BRCA1* exons 1–2 were characterized by intragenic breakpoints within 3.7 kb in intron 2 (Fig. 1). Telomeric breakpoints in *NBR2* and *LOC101929767* (*ΨBRCA1*), a partial *BRCA1* pseudogene, were associated with deletions of 6.6 kb and 36–37 kb, respectively. These findings were consistent with previous observations of recurrent deletions between *BRCA1* and adjacent loci [9].

**Table 1:** Summary of structural variants characterized by nanopore long-read genome sequencing.

Type	Gene (Transcript)	Affected exons	HGVS genomic nomenclature	Size (bp)	Reported ancestry	Number of individuals
Deletion	*BRCA1* (NM_007294.4)	1–2	NC_000017.11:g.43118925_43156395del	37,471	South East Asia	1
			NC_000017.11:g.43122618_43158674del	36,057	Britain	1
			NC_000017.11:g.43121293_43127941del	6649	Britain	1
		1–6	NC_000017.11:g.43101034_(43203943_43203947)del	102,910–102,914	North East Asia	1
		1–23	NC_000017.11:g.43023669_43131721del	108,053	Europe	1
		19	NC_000017.11:g.43054985_43060741del	5757	Europe	1
		21	NC_000017.11:g.43048723_43049232del	510	Eastern Europe	1
	*BRCA2* (NM_000059.4)	9–24	NC_000013.11:g.32330880_32382537del	51,658	Britain	1
		14–16	NC_000013.11:g.32349809_32360302del	10,494	Britain	1
		19–20	NC_000013.11:g.32366702_32374882del	8181	North East Asia	1
	*CHEK2* (NM_007194.4)	9–10	NC_000022.11:g.28696573_28701967del	5395	Western Europe	3
			NC_000022.11:g.28696638_28702825del	6188	Britain	2
	*PALB2* (NM_024675.4)	11–12	NC_000016.10:g.23606702_23615822del	9121	North East Asia	1
Duplication	*BRCA1* (NM_007294.4)	12	NC_000017.11:g.43078282_43084407dup	6126	Britain and Unknown	3

*bp* base pairs

Among founder populations, specific genetic variants make a considerable contribution to disease susceptibility. To explore the potential for nanopore GS to characterize founder haplotypes, we integrated read-based and statistical phasing using a reference haplotype panel from 2504 individuals sequenced as part of the 1000 Genomes Project Phase 3 [8]. For genomes with at least 20X average coverage (*n* = 13), read information alone allowed phasing for 77–92% of heterozygous SNVs, and longer reads were associated with larger haplotype blocks (Spearman correlation 0.78; Supplementary Fig. S1) [7]. Long reads spanning breakpoints of the British *BRCA1* founder duplication (ins6kbEx13) confirmed a 6126 bp tandem duplication in three unrelated individuals (Supplementary Fig. S2) [10]. Analysis of SNVs extending beyond the boundaries of the *BRCA1* ins6kbEx13 founder variant further defined a core 1.08 Mb haplotype shared between carriers.

**Fig. 2:**
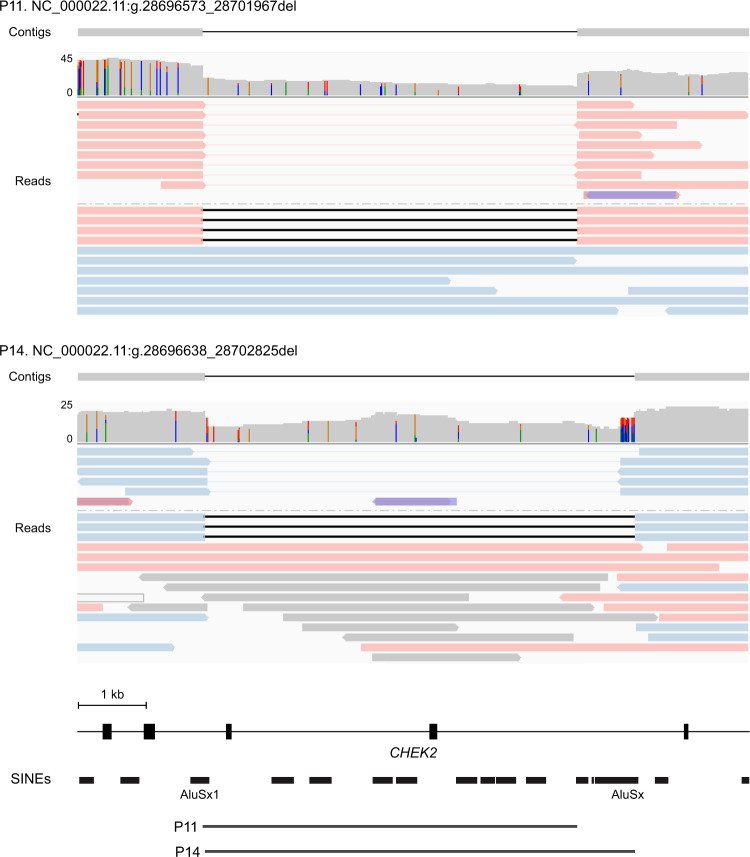
LRS reveals molecular heterogeneity of *CHEK2* exons 9–10 deletions. Read alignments and contigs derived by local assembly of variant supporting reads at the *CHEK2* locus. Reads are colored in blue and pink by haplotype inferred from integrated read-based and reference-guided phasing. Reads that could not be assigned to a haplotype are colored in gray. Local haploid assemblies were generated from reads supporting each variant to refine the breakpoints of the *CHEK2* del5395 European founder variant (P11) and the *CHEK2* del6188 variant (P14) in representative carriers. SINEs short interspersed nuclear elements. For clarity, mismatch bases and indels <50 bp are not shown.

Five individuals had deletions of *CHEK2* exons 9–10, characteristic of a 5395 bp deletion (del5395) estimated to account for 1% of breast cancers in Poland [11, 12]. LRS confirmed the *CHEK2* del5395 founder variant in three individuals; however, two related individuals had a larger 6188 bp deletion (del6188) with breakpoints in two Alu short interspersed nuclear elements with 71% sequence identity (Fig. 2 and Supplementary Fig. S2). Two base pair regions of microhomology at the breakpoints of the former suggest the del5395 and del6188 variants originated through distinct microhomology- and recombination-mediated mechanisms of formation, respectively. The *CHEK2* del5395 variant was associated with a core 1.26 Mb haplotype characterized by a rare SNV in *cis* located 100 kb upstream and specific to carriers of the del5395 founder variant (Supplementary Fig. S3). Together, these findings suggested that the del5395 and del6188 variants were unlikely to have arisen from the same subpopulation.

## Discussion

Advances in sequencing technologies have revealed a greater spectrum of heritable variation underlying human diversity and disease. Given the variable expressivity and genetic heterogeneity of breast cancer predisposition syndromes, multigene panel SRS has become widespread practice to identify families with an increased risk for disease. However, limitations of standard clinical assays for identifying complex genetic changes may underestimate the contribution of SVs to cancer susceptibility. Nanopore GS resolved 14 distinct copy number variants in high- and moderate-penetrance genes across 19 individuals with known breast cancer susceptibility. Our findings reveal unexpected allelic heterogeneity at the locus of *CHEK2*, and demonstrate the potential for LRS to characterize haplotype-resolved structural variation in personal genomes.

Characterizing the molecular heterogeneity of pathogenic variants in cancer susceptibility genes may inform estimates of individual cancer risk. Deletions of *BRCA1* exons 1–2 account for 10–15% of pathogenic copy number variants in *BRCA1* [13]. Consistent with previous reports, we identified recurrent deletions between 6.6 and 37 kb in three individuals with variable loss of *BRCA1* exons 1–2 and the 5’ region upstream. Targeted clinical testing could not reveal the extent of genetic loss. Among five individuals from four families with deletions of *CHEK2* exons 9–10, two related individuals were found to carry a 6188 bp deletion distinct from the del5395 Eastern European founder variant. Resolving the precise breakpoints of SVs may thus inform their molecular origins and natural history, and allow the development of customized confirmation assays for rapid and accurate carrier screening.

To a greater extent than deletions, the clinical interpretation of duplications remains challenging for SRS. Importantly, determining the location and orientation of duplications can inform the etiology of disease [14]. Nanopore sequencing accurately mapped the breakpoints of a tandem duplication and known founder variant, *BRCA1* ins6kbEx13, in three individuals. Despite sufficient read coverage, this variant was not identified by available SV callers, indicating a need for further development of SV detection methods using long reads. LRS has also shown potential to characterize cryptic, copy neutral and complex rearrangements whose clinical or functional significance is uncertain [15, 16]. These variants may remain undetected or unresolved by SRS, suggesting their contribution to cancer susceptibility may be underappreciated.

Using read-based and reference-guided phasing, we defined haplotypes shared between carriers of founder variants and identified rare alleles in *cis* that are likely to be identical by descent from a common ancestor. For diseases with common genetic aetiologies, chromosome-scale haplotyping may uncover alleles associated with causal variants in silent carriers who would otherwise go undetected [17]. The accuracy of reference-guided phasing depends on the composition and size of reference panels however, and many populations remain underrepresented in current population databases [18]. Therefore, large-scale efforts to characterize genetic variation across subpopulations of diverse genetic ancestries are needed.

The throughput and analytical validity of LRS have improved rapidly in the past several years with advances in Oxford Nanopore Technologies’ nanopore sequencing and Pacific Biosciences single-molecule, real-time sequencing. Recent library preparation and pore chemistries have allowed the sensitivity of SNV and indel calling from nanopore sequencing to exceed 99% in the coding genome [19]. The costs of nanopore GS, around $1500–$2000 CAD per sample, limit its wider clinical application compared to under $500 CAD for current clinical multigene panels. However, library multiplexing and PCR-free enrichment methods, including Cas9-mediated enrichment and computational selection by adaptive sampling, will enable cost-effective targeted nanopore sequencing whose throughput and accuracy could be comparable to multigene panel SRS [6, 20]. LRS thus offers a comprehensive testing strategy that may soon be readily adoptable in local diagnostic laboratories for routine testing of hereditary cancer susceptibility.

## Supplementary information

Supplementary Materials

## Data Availability

Raw sequencing data has been deposited in the European Genome-Phenome Archive as part of study EGAS00001005872. All scripts included in this work are available upon request.
