# Supplementary material for: Defining the heterogeneity of unbalanced structural variation underlying breast cancer susceptibility by nanopore genome sequencing
Source: Eur J Hum Genet. 2023 Feb 16;31(5):602–6. doi: 10.1038/s41431-023-01284-1 (PMC10172360; doi:10.1038/s41431-023-01284-1)
Supplement: Supplementary file 1 — Supplementary Materials [file 41431_2023_1284_MOESM1_ESM.pdf]

SUPPLEMENTARY METHODS

Genome sequencing was performed for DNA collected for the purpose of clinical or research genetic testing. Genomic DNA (gDNA) was isolated using DNeasy Blood and Tissue Kits (Qiagen, Germantown, USA). Size selection was performed using BluePippin (Sage Science) and gDNA was sheared to 10 kb using the Covaris g-TUBE. PCR-free DNA libraries were prepared according to standard protocols using the SQK-LSK109 or SQK-LSK110 Ligation Sequencing Kits (Oxford Nanopore Technologies, Oxford, UK). End-repair, A-tailing and adapter ligation were performed using the NEB Ultra II Kit (E7646A) and NEBNext quick ligase (E6056S) (New England Biolabs, Ipswich, USA). Sequencing was performed one sample per flow cell on R9.4.1 or R10.4.1 flow cells using the Oxford Nanopore Technologies PromethION. A DNase I nuclease flush (AM2222, ThermoFisher, Waltham, USA) was performed after 18 hours prior to loading remaining library for the same sample.

Base calling was performed using Guppy v4.0.11 and v5.0.7 using the high accuracy (r9.4.1_450bps_hac_prom) and super high accuracy (r9.4.1_450bps_sup_prom) models, respectively, and reads were aligned to the human reference genome version GRCh38 using mimimap2 version 2.15[1]. Small variant calling was performed according to best practices for joint calling using the PEPPER-Margin-DeepVariant pipeline (https://github.com/google/deepvariant/blob/r1.1/docs/trio-merge-case-study.md)[2]. Briefly, PEPPER-Margin-DeepVariant was run for each sample using the flags --phased and --gvcf, and joint calling was performed across the cohort using GLnexus and default configuration settings for DeepVariantWGS[3].

To characterise haplotypes associated with founder variants, haplotype inference was performed per chromosome using individual read-based phasing and reference haplotypes from the 1000 Genomes Project Phase 3. Reference haplotype panels for each chromosome were downloaded from http://ftp.1000genomes.ebi.ac.uk/vol1/ftp/data_collections/1000G_2504_high_coverage/working/20201028_3202_phased/. Joint variant calls were filtered to include single nucleotide variants (SNVs) at a minimum depth of 15X in all samples. Variants with missing genotypes were excluded. Reference-guided phasing was performed per individual based on joint genotypes and sample-specific read-based phasing from PEPPER-Margin-DeepVariant using SHAPEIT4[4]. Based on integrated phasing, reads aligning to the reference chromosome were assigned to two haplotypes using WhatsHap[5]. To refine structural variant breakpoints, local assemblies were

generated from reads supporting the variant using wtdbg2 with default parameters for preset3[6]. As some variants were not called by available SV callers Sniffles2 and cuteSV, reads supporting the variant were manually curated using the Integrative Genomics Viewer based on the presence of supplementary alignments, alignment gaps, and assigned haplotype[7,8].

REFERENCES

1. Li, H. Minimap2: Pairwise alignment for nucleotide sequences. *Bioinformatics* **34**, 3094–3100 (2018).
2. Shafin, K. *et al.* Haplotype-aware variant calling enables high accuracy in nanopore long-reads using deep neural networks. *bioRxiv* 2021.03.04.433952 (2021). doi:10.1101/2021.03.04.433952
3. Yun, T. *et al.* Accurate, scalable cohort variant calls using DeepVariant and GLnexus. *Bioinformatics* **36**, 5582–5589 (2021).
4. Delaneau, O., Zagury, J.-F., Robinson, M. R., Marchini, J. L. & Dermitzakis, E. T. Accurate, scalable and integrative haplotype estimation. *Nat. Commun. 2019 101* **10**, 1–10 (2019).
5. Martin, M. *et al.* WhatsHap: fast and accurate read-based phasing. *bioRxiv* 085050 (2016). doi:10.1101/085050
6. Ruan, J. & Li, H. Fast and accurate long-read assembly with wtdbg2. *Nat. Methods 2019 172* **17**, 155–158 (2019).
7. Sedlazeck, F. J. *et al.* Accurate detection of complex structural variations using single-molecule sequencing. *Nat. Methods* **15**, 461–468 (2018).
8. Jiang, T. *et al.* Long-read-based human genomic structural variation detection with cuteSV. *Genome Biol. 2020 211* **21**, 1–24 (2020).

SUPPLEMENTARY TABLES

Supplementary Table S1. Clinical testing information

ID	Family ID	Indication	Clinical Assay	Clinical Findings
P1	F1	Carrier	CMA	*BRCA1* exons 1-2 deletion
P2	F2	Index	Panel	*BRCA1* exons 1-2 deletion
P3	F3	Carrier	Panel	*BRCA1* exons 1-2 deletion
P4	F4	Index	Panel	*BRCA1* exons 1-6 deletion
P5	F5	Carrier	MLPA	*BRCA1* whole gene deletion
P6	F6	Carrier	MLPA	*BRCA1* c.5194-?_5277+?del
P7	F7	Index	Panel	*BRCA1* exon 21 deletion
P8	F8	Index	MLPA	*BRCA2* c.682-?_9256+?del
P9	F9	Carrier	CMA	13q31.1 (32352122_32359571)1X
P10	F10	Index	Panel	*BRCA2* exons 19-20 deletion
P11	F11	Carrier	Panel	*CHEK2* exons 9-10 deletion
P12	F12	Index	Panel	*CHEK2* exons 9-10 deletion
P13	F13	Index	Panel	*CHEK2* exons 9-10 deletion
P14	F14	Carrier	Panel	*CHEK2* exons 9-10 deletion
P15	F14	Carrier	Panel	*CHEK2* exons 9-10 deletion
P16	F15	Index	Panel	*PALB2* exons 11-12 deletion
P17	F16	Carrier	MLPA	*BRCA1* c.4186-?_4357+?dup
P18	F17	Carrier	CMA	*BRCA1* exon 13 duplication
P19	F18	Carrier	Panel	*BRCA1* exon 13 duplication

CMA, chromosome microarray; MLPA, multiplex ligation-dependent probe-based amplification.

Supplementary Table S2. Sequencing and variant information

ID	Guppy Version	Genome Coverage	N50	LRS Results	Method	Supporting Reads	Found by SV Calling
P1	4.0.11	29.5	12,096	NC_000017.11:g.43118925_43156395del	Contig	4	No
P2	5.0.7	17.9	23,457	NC_000017.11:g.43122618_43158674del	Contig	7	No
P3	4.0.11	24.9	12,016	NC_000017.11:g.43121293_43127941del	Contig	10	Yes
P4	4.0.11	19.0	10,560	NC_000017.11:g.43101034_(43203943_43203947)del	Reads	3	No
P5	4.0.11	21.5	15,596	NC_000017.11:g.43023669_43131721del	Contig	10	Yes
P6	4.0.11	23.5	14,683	NC_000017.11:g.43054985_43060741del	Contig	6	Yes
P7	4.0.11	15.7	14,722	NC_000017.11:g.43048723_43049232del	Contig	8	Yes
P8	4.0.11	17.5	14,503	NC_000013.11:g.32330880_32382537del	Contig	13	Yes
P9	4.0.11	26.8	15,715	NC_000013.11:g.32349809_32360302del	Contig	11	Yes
P10	4.0.11	13.1	13,897	NC_000013.11:g.32366702_32374882del	Contig	7	Yes
P11	4.0.11	30.4	13,561	NC_000022.11:g.28696573_28701967del	Contig	14	Yes
P12	4.0.11	36.5	15,313	NC_000022.11:g.28696573_28701967del	Contig	18	Yes
P13	4.0.11	34.0	11,263	NC_000022.11:g.28696573_28701967del	Contig	14	Yes
P14	4.0.11	17.8	10,233	NC_000022.11:g.28696638_28702825del	Contig	9	Yes
P15	5.0.7	14.8	18,463	NC_000022.11:g.28696638_28702825del	Contig	6	Yes
P16	4.0.11	26.9	6,567	NC_000016.10:g.23606702_23615822del	Contig	8	Yes
P17	4.0.11	21.6	15,995	NC_000017.11:g.43078282_43084407dup	Contig	13	No
P18	4.0.11	23.8	13,335	NC_000017.11:g.43078282_43084407dup	Contig	22	No
P19	5.0.7	14.3	16,321	NC_000017.11:g.43078282_43084407dup	Reads	4	No

SUPPLEMENTARY FIGURES

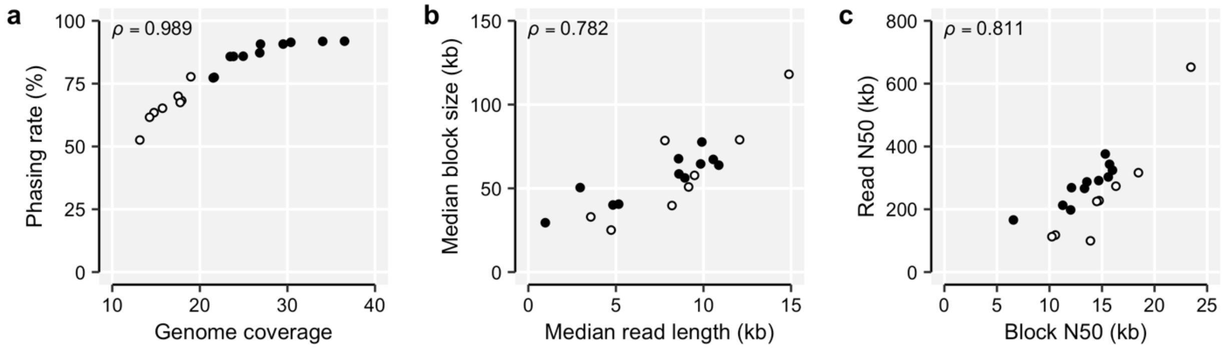

Supplementary Figure S1. The association of genome coverage and read length with phasing rate and block size. **a.** Association between average genome coverage and phasing rate, defined as the percent of heterozygous single nucleotide variants phased by read-based phasing. Genomes with < 20X average read coverage are shown as open circles. **b-c.** Association between read length and haplotype block size, measured according to median block size in kilobase pairs (kb) and median read length (**b**) and block N50 and read N50 (**c**).

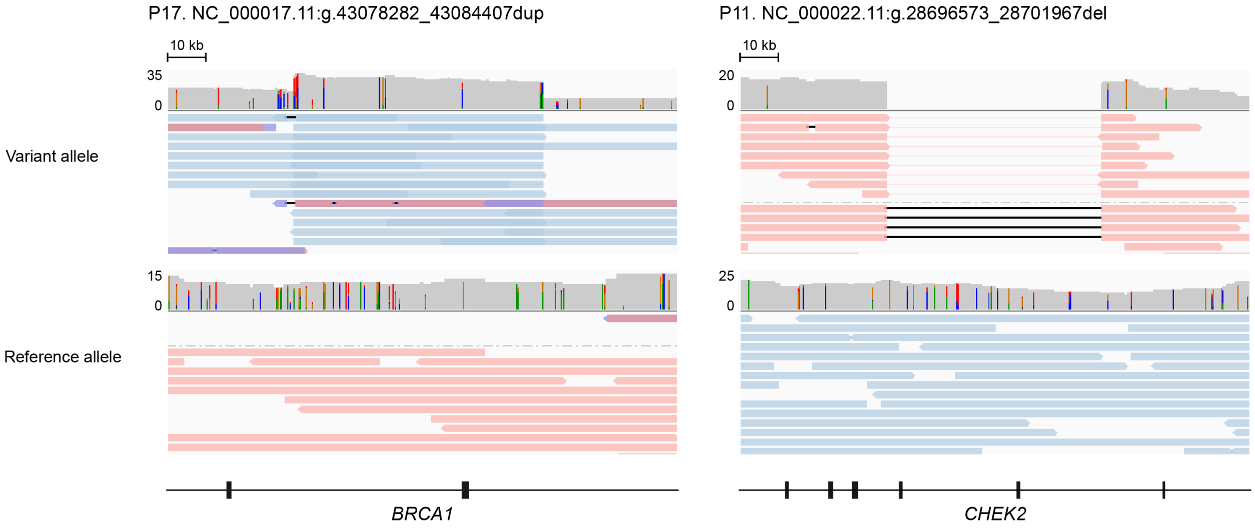

Supplementary Figure S2. Haplotype inference for founder variants by long reads and population haplotypes. Integrated read- and population-based phasing was performed using a reference haplotype panel to estimate chromosome-scale haplotypes. Reads assigned to haplotypes in representative carriers for the *BRCA1* ins6kbEx13 British founder duplication (P17) and *CHEK2* del5395 Eastern European founder deletion (P11). For clarity, mismatched bases and indels < 50 bp are not shown.

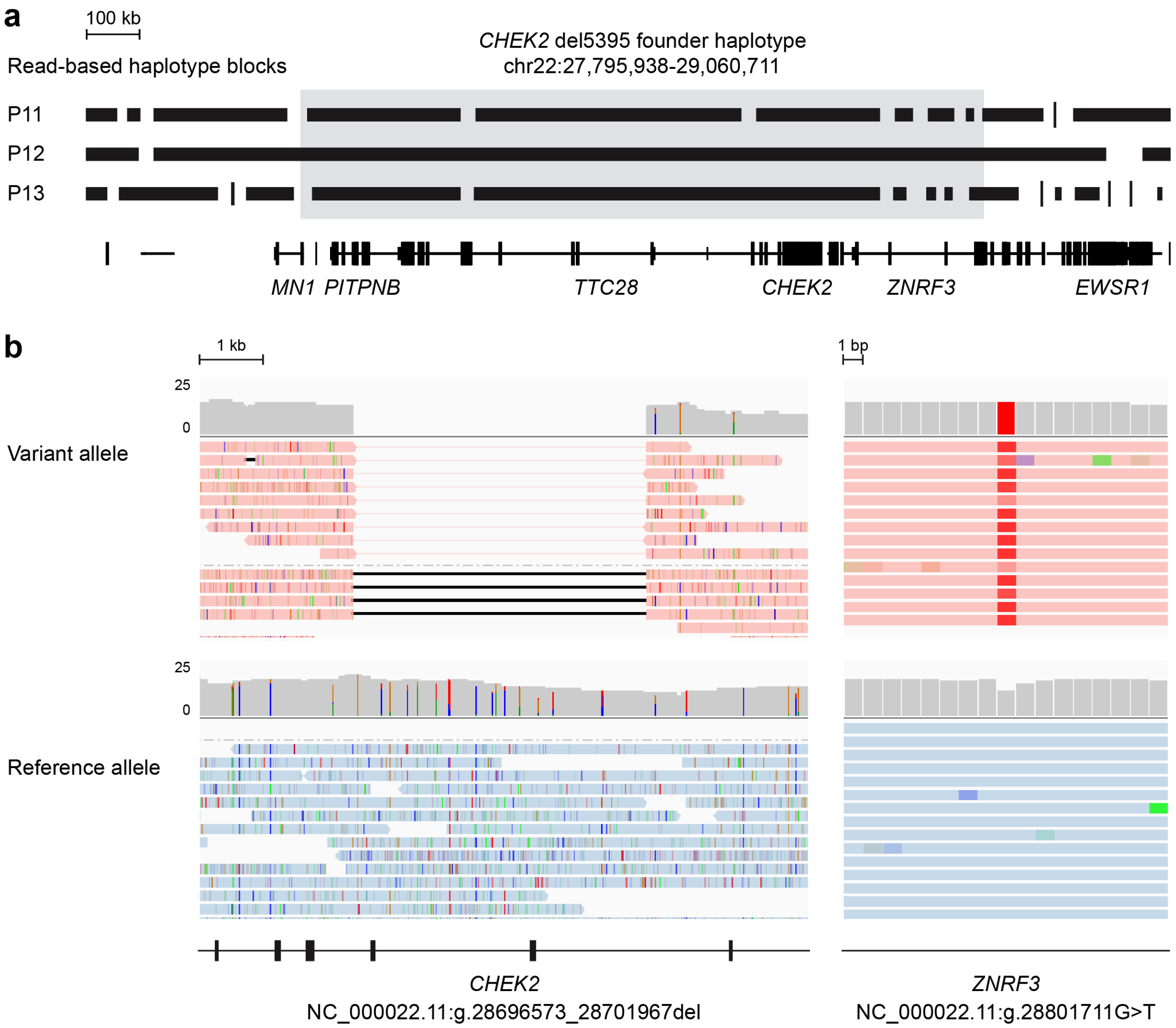

Supplementary Figure S3. Nanopore sequencing helps define a shared haplotype between carriers for the *CHEK2* del5393 founder variant. a. A core 1.26 Mb haplotype associated with the *CHEK2* del5395 founder deletion in three unrelated individuals defined by read- and population-based phasing. **b.** Haplotype-assigned reads at the loci of the del5395 variant and founder-specific NC_000022.11:g.28801711G>T (rs986514763) allele in a representative carrier (P11). For clarity, indels < 50 bp are not shown.
